# Prevalence, Severity, and Associated Factors of Postoperative Pain among Paediatric Patients Admitted to Paediatric Wards, Jimma University Medical Center, Ethiopia

**DOI:** 10.4314/ejhs.v35i3.4

**Published:** 2025-05

**Authors:** Samuel Negash Mankelkilot, Melkamu Berhane

**Affiliations:** 1 Department of Pediatrics and Child Health, Jimma University

**Keywords:** Pain severity, Postoperative pain, Associated factors

## Abstract

**Background:**

Pain is a stressful condition considered a global health problem, with children being the most vulnerable and underserved population. Inadequate management of pediatric postoperative pain (POP) results in increased suffering, morbidity, and mortality, prolonged hospital stays, and higher healthcare costs.

**Methods:**

A longitudinal prospective study design was employed using a structured questionnaire and checklist. The study population consisted of all pediatric patients who underwent surgical procedures and met the inclusion criteria. The consecutive sampling technique was used to enroll participants. Data entry and analyses were conducted using Epidata version 6.0 and Statistical Package for Social Sciences (SPSS) version 20.0. Bivariable and multivariable logistic regression analyses were performed to identify factors associated with the dependent variable.

**Results:**

A total of 154 postoperative pediatric patients participated in the study, yielding a 100% response rate. The mean age was 4.14 ± 4.19 years. The highest prevalence of overall POP and moderate-to-severe POP was observed at 6 hours postoperatively (n=129, 83.7%) and (n=60, 38.9%), respectively. Independent predictors associated with POP included incision size greater than 10 cm (AOR=8.73, 95% CI 1.07–71.02, p=0.043) and surgery duration ≥1 hour (AOR=2.02, 95% CI 1.01–4.03, p=0.045).

**Conclusion:**

The study revealed that the highest prevalence of moderate-to-severe POP occurred 6 hours post-surgery (60, 38.9%). Healthcare providers need to promptly assess and treat POP to reduce its consequences in this population.

## Introduction

Pain is defined by the International Association for the Study of Pain (IASP) as “an unpleasant sensory and emotional experience associated with actual or potential tissue damage or described in terms of such damage” (1). Pain is considered a global health problem, and severe pain is common after surgery, as surgery involves extensive tissue and bone trauma (2).

Pediatric pain is one of the most misunderstood, underdiagnosed, and undertreated medical issues worldwide, especially in developing countries. Similarly, postoperative pain (POP) is often undertreated, even in developed countries. Pediatric surgical patients are particularly at risk for inadequate pain management (3). Several studies on POP reveal a high prevalence, ranging from 30% to 80% (4, 5, 6). In Ethiopia, although data on pediatric POP is scarce, a cross-sectional study in northwestern Ethiopia showed a high prevalence of moderate-to-severe POP (40.5%) (7).

Inadequately treated or untreated pain is a significant concern for pediatric surgical patients, as it leads to increased suffering, higher morbidity and mortality, longer hospital stays, and increased healthcare costs (3). For effective POP management, healthcare professionals need valid tools to assess and classify pain severity, which will guide appropriate treatment (8). However, pain assessment in infants and young children is challenging, primarily because they cannot easily express or describe pain intensity, making it difficult for professionals to manage their pain effectively (7).

Other reasons for inadequate POP management include the lack of routine pain assessment and documentation, the absence of written POP assessment and management protocols, deficiencies in pain management education, and a lack of effective analgesic medications (9, 10, 11, 12, 13, 14, 15).

Several factors have been associated with POP, including sociodemographic characteristics, surgical factors, anesthetic techniques, and the analgesic drugs used during surgery (16). This study aimed to assess the prevalence, severity, and factors associated with POP in pediatric postoperative patients.

## Methods and Materials

**Study area, period, and design**: A longitudinal prospective study was conducted at the pediatric wards of Jimma University Medical Centre (JUMC), Jimma, Southwest Ethiopia, from July 15 to November 15, 2023.

**Population**: The source population consisted of all pediatric patients who underwent surgical procedures and were admitted to the pediatric wards of JUMC during the study period. The study population included all pediatric patients who underwent surgery and met the inclusion criteria.

**Inclusion and exclusion criteria**: Pediatric surgical inpatients aged 2 months to 15 years, who underwent surgery during the study period and whose parents/caregivers provided consent, were included. Exclusion criteria included patients with preexisting neurological deficits, those who underwent outpatient surgery, and those whose parents/caregivers declined participation.

**Sample size determination**: Sample size was determined using a single population proportion formula, based on the following assumptions: p = 40.5% (7), 95% confidence interval, and a 5% margin of error.

After calculating, the initial sample size was approximately 370. Since the source population was fewer than 10,000, the final sample size was adjusted using the finite population correction formula, resulting in an estimated sample size of 154 after accounting for a 10% non-response rate.

**Sampling methods**: The consecutive sampling technique was employed to enroll participants who met the inclusion criteria until the required sample size was reached.

**Data collection instruments**: A structured questionnaire was developed based on a review of the relevant literature. The Face, Leg, Activity, Cry, and Consolability (FLACC) scale was used to assess pain levels. The FLACC scale is a reliable and valid observational tool for assessing pain in infants and children (17). It provides a score of 0–10, with severity levels categorized as no pain (0), mild pain (1–3), moderate pain (4–6), and severe pain (7–10) (19, 20).

**Data collection procedures**: To minimize bias, data was collected by trained nurses not involved in the care of the patients. The structured questionnaire was used to collect demographic and clinical data, including details of the surgery, anesthesia, analgesia, and pain severity as documented using the FLACC scale at 2, 6, 12, 24, and 72 hours post-surgery.

**Data analysis**: The collected data was cleaned, coded, and entered into EpiData version 6.0, then exported to SPSS version 20.0 for analysis. Descriptive statistics, including frequency distributions, tables, and summary measures, were computed. Bivariate logistic regression was performed to identify variables associated with POP. Variables with a p-value ≤ 0.25 in the bivariate analysis were included in the multivariable regression model. Variables with a p-value < 0.05 were considered statistically significant.

**Ethical consideration**: Ethical approval was obtained from the Institutional Review Board (IRB) of Jimma University (Ref. No: JUIH/IRB/604/23). Permission to conduct the research was granted by the hospital administration. Written consent was obtained from each postoperative patient or caregiver.

## Results

**Sociodemographic characteristics**: A total of 154 patients participated, with a 100% response rate. The majority of respondents were male (n=106, 68.8%) and under one year of age (n=51, 33.1%). The mean age was 4.14 ± 4.19 years, ranging from 2 months to 15 years (Table 1).

**Table 1:** Socio-demographic characteristics of the study participants at JUMC, Jimma, Ethiopia, 2023(n=154)

Variables	Categories	Frequency	Percent (%)
**Age**	2months to 1year	51	33.1
	1-3 years	27	17.5
	3-6 years	28	18.2
	6-12years	35	22.7
	12-15 years	13	8.4
**Sex**	Male	106	68.8
	Female	48	31.2

**Surgery-related factors**: Most participants underwent surgery with general anesthesia (n=143, 92.9%) and on an elective basis (n=99, 64.3%). The most common surgical site was the abdomen (n=96, 62.3%), and the most frequent procedure was laparotomy (n=28, 18.2%) (Table 2).

**Table 2:** Surgery related factors of the study participants who underwent surgical intervention at JUMC, Jimma, southwest Ethiopia, 2023(n=154)

Variables	Categories	Frequency	Percent (%)
**Type of surgery**	Elective	99	64.3
	Emergency	55	35.7
**Anatomical site of surgery**	Abdominal	96	62.3
	Urologic	28	18.2
	Neurologic	12	7.8
	ENT	11	7.1
	Extremities	4	2.6
	Cardiothoracic	3	1.9
**Type of procedures**	Laparatomy	61	39.6
	Herniorrhaphy	15	9.7
	Hypospadial repair	15	9.7
	Incision, drainage and Debridment	11	7.1
	Excisional/incisional biopsy	7	4.5
	PSARP*	6	3.9
	Pyloromyotomy	5	3.2
	VPS**	5	3.2
	Thoracostomy tube	3	1.9
	Others	26	16.9
**Length of incision**	<5cm	63	40.9
	5-10cm	38	24.7
	>10cm	16	10.4
	Unestimated	37	24.0
**Duration of surgery**	<1hr	69	44.8
	≥1hr	85	55.2
**Type of anesthesia**	General anesthesia	143	92.9
	Spinal and local anesthesia	11	7.1

Others: Nephrectomy (3), tethered cord release (3), orchidopexy (3), cranioplasty (3), cordal correction (3), meningocele repair (3), vesicostomy (3), high ligation of hernia sac (2) and urethrocutaneous fistula repair (3)

* Posterior sagital anorectoplasty

** Ventriculo peritoneal shunt

**Postoperative analgesia management**: Regarding POP management, most participants (110, 71.4%) received paracetamol rectally, with this being the most common route of analgesic administration (90, 60.4%) (Table 3).

**Table 3:** Analgesia related factors of the study participants who underwent surgical intervention at JUMC, Jimma, south west Ethiopia, 2023(n=154)

Variables	Categories	Frequency	Percent (%)
**Types of analgesics**	Morphine	6	3.9
	Tramadol	7	4.5
	Diclofenac	9	5.8
	Paracetamol	110	71.4
	Two drug	22	14.3
**Doses of analgesics**	Under dose	2	18.2
	Appropriate dose	91	59.1
	Overdose	35	22.7
**Routes of analgesics prescribed**	Intramuscular	8	5.2
	Oral	15	9.7
	Intravenous	16	10.4
	Rectal	93	60.4
	More than one route	22	14.3

**Figure 1:**
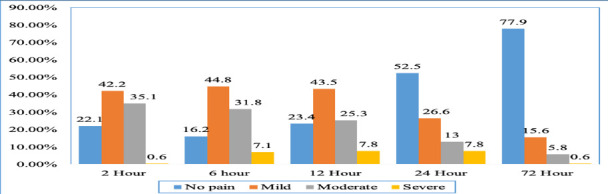
Distribution of pain severity at different time point after surgery among pediatric patients at Jimma University Medical Centre, 2023

**Prevalence and Severity of POP**: The highest prevalence of POP occurred 6 hours after surgery (129, 83.8%), with the highest prevalence of severe POP observed at 12 and 24 hours post-surgery (12, 7.8% each) (Figure 1).

**Factors Associated with POP**: Bivariate analysis at 6 hours post-surgery identified several variables with a p-value ≤ 0.25, including patient sex, surgery type, incision size, and surgery duration (Table 4). Multivariate analysis revealed that incision size >10 cm (AOR=8.7, 95% CI 1.07–71.02, p=0.043) and surgery duration ≥1 hour (AOR=2.02, 95% CI 1.01–4.03, p=0.045) were significantly associated with POP (Table 5).

**Table 4:** Bivariate logistic regression analysis of factors associated with POP among paediatric patients who underwent surgery at Jimma University Medical Centre, 2023

Variables	Category	Outcome Variables		COR 95%CI	p-value
		Moderate-severe pain (n) (%)	No pain-mild (n) (%)		
**Sex**	Male	57 (53.8)	49 (46.2)	1	0.027*
	Female	35 (72.9)	13 (27.1)	2.31(1.10-4.86)	
**Type of surgery**	Elective	55 (55.6)	44 (44.4)	1	0.157*
	Emergency	37 (67.3)	18 (32.7)	1.64 (0.83-3.27)	
**Anatomical site of surgery**	Abdominal	66 (68.8)	30 (31.3)	0.73 (0.073-7.34)	0.792
	ENT	2 (18.2)	9 (81.8)	0.07 (0.005-1.14)	0.062*
	Neurologic	6 (50.0)	6 (50.0)	0.33 (0.027-4.18)	0.395
	Cardiothoracic	2 (66.7)	1 (33.3)	0.66(0.025-18.06)	0.810
	Urologic	13 (46.4)	15 (53.6)	0.289(0.027-3.13)	0.307
	Extremities	3 (75.0)	1 (25.0)	1	1
**Incisional size**	<5 cm	37 (58.7)	26 (41.3)	1	1
	5-10 cm	25 (93.8)	13 (34.2)	1.35 (0.58-3.12)	0.481
	>10 cm	15 (93.8)	1 (6.3)	10.54 (1.31-84.83)	0.027*
	Unestimated	15 (40.5)	22 (59.5)	0.48 (0.21-1.09)	0.081*
**Types of anesthesia**	General	88 (61.5)	55 (38.5)	4.00 (0.75-21.33)	0.105*
	Spinal	2 (50.0)	2 (50.0)	2.50 (0.19-32.19)	0.48
	Local	2 (28.6)	5 (71.4)	1	1
**Types of analgesics**	Tramadol	4(57.1)	3(42.9)	0.62(0.09-3.56)	0.59
	Diclofenac	5(55.6)	4(44.4)	0.58(0.12-2.86)	0.51
	Morphine	5(83.3)	1(16.7)	2.33(0.23-23.91)	0.47
	Paracetamol	63(57.3)	47(42.7)	0.63(0.36-1.66)	0.34
	Two drug combination	15(68.2)	7(31.8)	1	1
**Dose of analgesic**	Under dose	17 (60.7)	11 (39.3)	0.71 (0.25-2.00)	0.52
	Appropriate dose	51 (56.0)	40 (44.0)	0.58 (0.25-1.33)	0.20*
	Over dose	24 (68.6)	11 (31.4)	1	1
**Routs of analgesics prescribed**	Intravenous	9 (56.2)	7 (43.8)	1	1
	Intramuscular	6 (75.0)	2 (25.0)	0.60 (0.16-2.28)	0.45
	Oral	6 (40.0)	9 (60.0)	1.40 (0.22-8.77)	0.72
	Rectal	56 (60.2)	37 (39.8)	0.31 (0.08-1.22)	0.09*
	More than one route	15 (68.2)	7 (31.8)	0.71 (0.26-1.89)	0.49
**Duration of surgery**	<1hr	33 (47.8)	36 (52.2)	1	0.033*
	≥ 1hr	59 (69.4)	26 (30.6)	2.47 (1.28-4.79)	

* Candidate variables for multivariate logistic regression

**Table 5:** Multivariable logistic regression model to identify factors associated with POP among pediatric patients who underwent surgery at Jimma University Medical Center, 2023

Variables	Category	Outcome Variables		AOR 95%CI	p-value
		Moderate-severe pain (n) (%)	Nopain-mild (n) (%)		
**Sex**	Male	57 (53.8)	49 (46.2)	1	0.11
	Female	35 (72.9)	13 (27.1)	1.95 (0.85-4.48	
**Type of surgery**	Elective	55 (55.6)	44 (44.4)	1	0.89
	Emergency	37 (67.3)	18 (32.7)	1.06 (0.42-2.68)	
**Anatomical site of surgery**	Abdominal	66 (68.8)	30 (31.3)	0.33 (0.02-6.73)	0.46
	ENT	2 (18.2)	9 (81.8)	0.05 (0.02-1.31)	0.07
	Neurologic	6 (50.0)	6 (50.0)	0.22 (0.01-5.25)	0.35
	Cardiothoracic	2 (66.7)	1 (33.3)	0.45 (0.01-29.26)	0.69
	Urologic	13 (46.4)	15 (53.6)	0.35 (0.02-7.91)	0.51
	Extremities	3 (75.0)	1 (25.0)	1	1
**Incisional size**	**<5 cm**	**37 (58.7)**	**26 (41.3)**	**1**	**1**
	**5-10 cm**	**25 (93.8)**	**13 (34.2)**	**1.25 (0.53-2.92)**	**0.61**
	**>10 cm**	**15 (93.8)**	**1 (6.3)**	**8.73 (1.07-71.21)**	0.043**
	**Unestimated**	**15 (40.5)**	**22 (59.5)**	**0.49 (0.21-1.14)**	**0.10**
**Types of anesthesia**	General	88 (61.5)	55 (38.5)	6.12 (0.67-38.78)	0.054
	Spinal	2 (50.0)	2 (50.0)	4.68 (0.29-74.79)	0.27
	Local	2 (28.6)	5 (71.4)	1	1
**Dose of analgesic**	Under dose	17 (60.7)	11 (39.3)	0.67 (0.20-2.29)	0.53
	Appropriate dose	51 (56.0)	40 (44.0)	0.61 (0.24-1.59)	0.316
	Over dose	24 (68.6)	11 (31.4)	1	1
**Routs of analgesics prescribed**	Intravenous	9 (56.2)	7 (43.8)	1	1
	Intramuscular	6 (75.0)	2 (25.0)	0.86 (0.08-9.22)	0.90
	Oral	6 (40.0)	9 (60.0)	2.08 (0.32-13.42)	0.44
	Rectal	56 (60.2)	37 (39.8)	2.18 (0.52-9.16)	0.28
	More than one route	15 (68.2)	7 (31.8)	1.83 (0.34-9.83)	0.48
**Duration of surgery**	**<1hr**	**33 (47.8)**	**36 (52.2)**	**1**	0.045**
	**≥ 1hr**	**59 (69.4)**	**26 (30.6)**	**2.02 (1.01-4.03)**	

** The independent variables having statistically significant association with the dependent variable

## Discussion

This study found the highest prevalence of both overall and moderate-to-severe POP at 6 hours post-surgery, consistent with findings from a study conducted in Gonder, where moderate-to-severe POP was prevalent in 36.6% of patients (7). The high prevalence of pain suggests that many children continue to experience unacceptable pain levels in the immediate postoperative period. The need for prompt POP assessment and management, particularly in the transition period after anesthesia wears off, is critical.

Our study showed a higher prevalence of moderate-to-severe POP compared to other studies, such as those in South Africa (22%), Northeastern Thailand (27%), Austria (25%), and France (10.9%) (22, 23, 24, 25). This discrepancy could be due to differences in pain management practices, including the use of multimodal analgesia.

Despite the decrease in prevalence over time, more than 20% of patients still experienced pain 72 hours after surgery, indicating a significant unmet need for effective pain management.

Incision size >10 cm and surgery duration >1 hour were identified as significant risk factors for POP. Larger incisions likely cause more tissue damage, while longer surgeries lead to prolonged pain postoperatively. These findings emphasize the need for heightened attention to pain management for patients with these risk factors.

In conclusion, the prevalence of POP is high in this setting. Efforts should be made to improve the assessment and management of POP to reduce its magnitude and the associated short- and long-term consequences.
